# PCNA antagonizes cohesin-dependent roles in genomic stability

**DOI:** 10.1371/journal.pone.0235103

**Published:** 2020-10-19

**Authors:** Caitlin M. Zuilkoski, Robert V. Skibbens

**Affiliations:** Department of Biological Sciences, Lehigh University, Bethlehem, Pennsylvania, United States of America; Texas A&M University College Station, UNITED STATES

## Abstract

PCNA sliding clamp binds factors through which histone deposition, chromatin remodeling, and DNA repair are coupled to DNA replication. PCNA also directly binds Eco1/Ctf7 acetyltransferase, which in turn activates cohesins and establishes cohesion between nascent sister chromatids. While increased recruitment thus explains the mechanism through which elevated levels of chromatin-bound PCNA rescue *eco1* mutant cell growth, the mechanism through which PCNA instead worsens cohesin mutant cell growth remains unknown. Possibilities include that elevated levels of long-lived chromatin-bound PCNA reduce either cohesin deposition onto DNA or cohesin acetylation. Instead, our results reveal that PCNA increases the levels of both chromatin-bound cohesin and cohesin acetylation. Beyond sister chromatid cohesion, PCNA also plays a critical role in genomic stability such that high levels of chromatin-bound PCNA elevate genotoxic sensitivities and recombination rates. At a relatively modest increase of chromatin-bound PCNA, however, fork stability and progression appear normal in wildtype cells. Our results reveal that even a moderate increase of PCNA indeed sensitizes cohesin mutant cells to DNA damaging agents and in a process that involves the DNA damage response kinase Mec1(ATR), but not Tel1(ATM). These and other findings suggest that PCNA mis-regulation results in genome instabilities that normally are resolved by cohesin. Elevating levels of chromatin-bound PCNA may thus help target cohesinopathic cells linked that are linked to cancer.

## Introduction

During S phase, the cellular genome duplicates and each sister chromatid becomes tethered together to ensure proper inheritance of the genome during mitosis. Sister chromatid tethering is maintained by cohesin, a complex comprised of Smc1, Smc3, and Mcd1/Scc1/RAD21 along with auxiliary subunits Pds5, Scc3/Irr1, Rad61/WAPL and, in vertebrate cells, Sororin [1–7]. Cohesin deposition onto DNA requires the cohesin loader, comprised of Scc2 and Scc4, which functions through a large part of the cell cycle but is essential during S phase for cohesins to participate in sister chromatin tethering [8, 9]. Deposition onto chromatin, however, is not sufficient for cohesion. Eco1/Ctf7 (herein Eco1) is an acetyltransferase that converts chromatin-bound cohesins, through acetylation of Smc3 cohesin subunit, to a tethering competent state [3, 10–13]. Early studies coupled this process of cohesion establishment to the DNA replication factor PCNA [10]. PCNA directly binds and recruits Eco1 to the DNA replication fork [14–16], suggesting that establishment is coordinated with numerous processes (histone deposition, chromatin remodeling, DNA repair and translesion synthesis) directed by PCNA and regulated through PCNA post-translational modifications [17–20]. Elevated recruitment to the DNA replication fork thus provides a plausible explanation for PCNA-dependent suppression of *eco1* mutant cell growth defects [10, 15]. In support of this model, genetic fusing of *ECO1* and *POL30* (PCNA) restores cohesion defects otherwise present in *eco1* PIP box mutants [21]. The repertoire of DNA replication factors (Chl1, Ctf4, Ctf18, MCM2-7, for example) implicated in cohesion establishment regulation has grown substantially [15, 22–36], highlighting the fundamental and highly conserved nature through which cohesion establishment is obligatorily coordinated with DNA replication.

Further complicating the relationship between PCNA, Eco1 and cohesins are the additional roles played beyond sister chromatid tethering [1, 5, 10, 21, 37–48]. For instance, cohesin functions in both DNA replication restart during S phase and high fidelity DNA repair after S phase [49–51]. Cohesin is recruited to stalled DNA replication forks during S phase, as well as to rDNA and telomeric regions that experience prescribed pauses during replication [52–54]. Cohesin is similarly recruited to sites of DNA damage to promote high fidelity repair by ensuring proximity to the undamaged sister template [50, 55, 56]. Intriguingly, once recruited to stalled replication forks and sites of DNA damage, cohesin must ultimately dissociate from DNA to promote replication fork restart [57]. Thus, cohesin and PCNA appear to play highly coordinated roles in DNA metabolism.

PCNA impact on cohesin complexes, however, is quite complex. Deletion of the PCNA-dissociation factor *ELG1* (*elg1Δ*), or simply overexpression of PCNA (PCNA^OE^), result in long-lived retention and increased levels of chromatin-bound PCNA which in turn rescues *eco1* and *pds5* mutant cell cohesion defects and viability [10, 15, 32–34, 58–63]. Conversely, *elg1Δ* and PCNA^OE^ each exacerbate the growth defects exhibited by cohesin (*mcd1*, *smc1* and *smc3*) mutant cells [15, 32, 33, 35]. The mechanism through which elevated levels of chromatin-bound PCNA antagonize cohesin mutant cell growth remains unknown, highlighting a critical deficit in current descriptions of cohesin regulation. In this current study, we confirm the adverse impact that PCNA^OE^ exerts on cohesin mutant strains, even while having no overt adverse effect in wildtype cells. We then test multiple mechanisms through which the long-lived retention, and increased levels, of chromatin-bound PCNA exacerbates cohesin mutant cells: models that include competition for DNA access, changes in cohesin acetylation states, and increased sensitivity to genotoxic agents.

## Materials and methods

### Yeast strains and bacterial plasmids

All reagents, yeast strains, and bacterial plasmids are listed in S1–S3 Tables, respectively. All yeast strains used in this study were performed in a W303 background strain unless otherwise noted (S2 Table). V5 tagged Smc3 strains were created using EU3430-9A, generously given by Drs. Vincent Guacci and Douglas Koshland. Primers used to amplify the SMC3:V5:HIS region, primers are (forward primer) 5’-TTAACGCGGTTGATTTCTACTTTCCAAAAGGTTTCTGAAAA-3’ and (reverse primer) 5’-TAGCTCTGATTCTGACTCTAACTCCAGTTCGGACTCCGTATCGGATTCCAGTTCAGATTC-3’. The resulting PCR product was transformed into a wildtype W303 background strain (YCZ044) to produce YCZ249. YCZ249 was mated with YCZ262 and a genetic cross was used to obtain YCZ280. YCZ280 was mated with YBS2012 to obtain YCZ407, YCZ408, YCZ425, and YCZ428 (see S2 Table). A CEN vector plasmid or *2μ POL30* plasmid were transformed into YCZ147 and YCZ144 to obtain the resulting yeast strains: YCZ477, YCZ465, YCZ474, and YCZ225. YCZ559, YCZ561, YCZ563, and YCZ565 were created by transforming either a CEN vector plasmid or *2μ POL30* plasmid into K5824 and K6013, respectively, generously given by Dr. Vincent Guacci. A CEN vector plasmid or *2μ POL30* plasmid were transformed into YMM433 and YMM435 to obtain the resulting yeast strains (YCZ530, YCZ532, YCZ534, YCZ536). A CEN vector plasmid or *2μ POL30* plasmid were transformed into YDM884 and DLY285 to obtain the resulting yeast strains (YCZ567 through YCZ578). A pGADT7 vector plasmid or a *2μ ADH AD*:*HA*:*POL30* plasmid was transformed into yeast strains K699, K5824, and K6013 to generate resulting yeast strains (YCZ662 through YCZ673). *2μ POL30* plasmid was transformed into yeast strains K5824 and K6013 to generate resulting yeast strains (YCZ559, YCZ561, YCZ563, YCZ565). Primers (forward primer) 5’- AGAGAAGGTTTTCCAATGAAAAGGCACGTGTCTTTATCTGATATATTGACAGGAAATAAGCGGATCCCCGGGTTAATTAA-3’and (reverse primer) 5’- ATTTCCCCGCACTACCATGCTATATTTATTATACATACGTGTTCCTGTAACGATGCACGCGAATTCGAGCTCGTTTAAAC-3’ were used to generate strains harboring *ELG1*::*KAN* as previously described [64]. The resulting PCR product was transformed into K5824 to obtain the yeast strains YCZ706 and YCZ707. YCZ143 was mated to K6013 to obtain YCZ702 and a genetic cross was used to obtain YCZ728 and YCZ729. CZ421 was mated to CZ778, generously given by Dr. Gregory Lang, to obtain CZ799 and a genetic cross was used to determine the genetic interaction between *msh3Δ*, *elg1Δ*, and *mcd1-1* alleles. Strains and plasmids are available upon request.

### Dilution plating

Cells were grown at 23° overnight in either YPD or selection media, normalized by OD600, and then plated in 10-fold serial dilutions at the indicated temperatures on YPD medium or selective medium. Cells grown on YPD medium or selective medium supplemented with methyl methanesulfonate (Sigma) or hydroxyurea (Sigma) were treated in a similar manner.

### Smc3 chromatin binding assay

Log phase yeast strains were grown to 0.6 OD600 and synchronized in G1 by exposing cells to fresh media supplemented with alpha factor at the permissive temperature of 23°C for three hours. The resulting G1 synchronized cells were washed, cultures split in half and maintained for three hours at 34°C or 37°C for 3 hours. Nocodazole arrested cells were harvested to assess Smc3 chromatin binding levels, based on modifications of previously described protocols [65]. Cell cultures were normalized to OD600 between 0.3–0.6, pelleted and washed with 1.2 M Sorbitol. Cells were resuspended in CB1 buffer (50 mM Sodium citrate, 1.2 M Sorbitol, 40 mM EDTA, pH 7.4) and spheroblasted. Spheroblasted cells were washed, resuspended in 1.2 M Sorbitol, and frozen in liquid nitrogen. Cells were thawed on ice and supplemented with Lysis buffer (500 mM Lithium Acetate, 20 mM MgSO4, 200 mM HEPES, pH 7.9), protease inhibitor cocktail (AEBSF, 1,10-Phenanthroline, Pepstatin A, E-64) (Sigma), and TritonX-100. Lysates were centrifuged at 12,000g for 15 minutes and soluble and containing chromatin bound fractions collected and denatured using 2X Laemelli buffer. Whole cell extracts, supernatant, and pellet were resolved by SDS-PAGE and analyzed by Western blot using anti-V5 (1:40,000) (Invitrogen) with goat anti-mouse HRP (1:40,000) (Bio-Rad), anti-PGK (1:20,000) (Novex) with goat anti-mouse HRP (1:40,000) (Bio-Rad), or anit-H2B (1:80,000) (Abcam) with goat anti-rabbit HRP (1:40,000) (Bio-Rad) and ECL prime (GE Healthcare) for visualization.

### Smc3 acetylation assay

Log phase yeast strains were grown to 0.6 OD600 and synchronized in G1 by exposing cells to fresh media supplemented with alpha factor at the permissive temperature of 23°C for three hours. The resulting G1 synchronized cells were washed, cultures split in half and maintained for three hours at 34°C or 37°C. Nocodazole arrested cells were harvested to assess Smc3 acetylation, with additional modifications as previously described [65]. Cell cultures were normalized to an OD600 between 0.3–0.6. Cells were washed in sterile water, then resuspended in sterile water prior to freezing at -80°C. Frozen pellets were extracted by the addition of IPH50 buffer (50mM Tris pH 7.8, 150mM NaCl, 5mM EDTA, 0.5% IGEPAL 630 (Sigma), 10mM Sodium Butyrate, 1mM DTT) and glass beads prior to bead beating (BioSpec). Cell lysates were supplemented with IPH50 buffer and protease inhibitor cocktail (AEBSF, 1,10-Phenanthroline, Pepstatin A, E-64) (Sigma), centrifuged at 15,000rpm for 20 minutes, and the pellet washed with sterile water before centrifugation at 15,000rpm for 10 minutes. The resulting chromatin fraction was supplemented with SBIIA buffer (0.5M Tris pH 9.4, 6% Sodium Dodecyl Sulfate before a 10-minute incubation at 50°C. SBII buffer (50% glycerol supplemented with bromophenol blue) and 1M DTT buffer was added to the cell lysates followed by a 5-minute incubation at 65°C. Whole cell protein samples were resolved by SDS-PAGE electrophoresis and analyzed by Western blot using anti-V5 (1:40,000) (Invitrogen) with goat anti-mouse HRP (1:40,000), anti-PGK (1:20,000) (Novex) with goat anti-mouse HRP (1:40,000) (Bio-Rad) or by anti-Smc3 K112/K113 Acetylation (1:1,000, gift from Dr. Katsuhiko Shirahige) in combination with goat anti-mouse HRP (1:10,000) (Bio-Rad) and ECL prime (GE Healthcare) for visualization.

### PCNA chromatin binding assay

Log phase yeast strains were normalized to OD600 between 0.3–0.6, pelleted and washed with 1.2 M Sorbitol. Cells were resuspended in CB1 buffer (50 mM Sodium citrate, 1.2 M Sorbitol, 40 mM EDTA, pH 7.4) and spheroblasted. Spheroblasted cells were washed, resuspended in 1.2 M Sorbitol, and frozen in liquid nitrogen. Cells were thawed on ice and supplemented with Lysis buffer (500 mM Lithium Acetate, 20 mM MgSO4, 200 mM HEPES, pH 7.9), protease inhibitor cocktail (AEBSF, 1,10-Phenanthroline, Pepstatin A, E-64) (Sigma), and TritonX-100. Lysates were centrifuged at 12,000g for 15 minutes and soluble and containing chromatin bound fractions collected and denatured using 2X Laemelli buffer. Whole cell extracts, supernatant, and pellet were resolved by SDS-PAGE and analyzed by Western blot using anti-PCNA (1:1,000) (Abcam) with rabbit anti-mouse HRP (1:40,000) (Bio-Rad), anti-PGK (1:20,000) (Novex) with goat anti-mouse HRP (1:40,000) (Bio-Rad), or anit-H2B (1:80,000) (Abcam) with goat anti-rabbit HRP (1:40,000) (Bio-Rad) and ECL prime (GE Healthcare) for visualization.

### Rad53 phosphorylation assay

Log phase yeast strains were grown to 0.6 OD600 and synchronized in G1 by exposing cells to fresh media supplemented with alpha factor at the permissive temperature of 23°C for three hours. The resulting G1 synchronized cells were washed, and half of the wildtype cell culture was exposed to 0.05% MMS to induce Rad53 phosphorylation. Cultures were then maintained for three hours at 37°C. Nocodazole arrested cells were harvested to assess Rad53 phosphorylation, with additional modifications as previously described [65]. Cell cultures were normalized to an OD600 between 0.3–0.6. Cells were washed in sterile water, then resuspended in sterile water prior to freezing at -80°C. Frozen pellets were extracted by the addition of Trichloroacetic acid (TCA) and glass beads prior to bead beating (BioSpec). Cell lysates were supplemented with TCA, centrifuged at 15,000rpm for 20 minutes, and the pellet washed with sterile water before centrifugation at 15,000rpm for 10 minutes. The resulting chromatin fraction was supplemented with SBIIA buffer (0.5M Tris pH 9.4, 6% Sodium Dodecyl Sulfate before a 10-minute incubation at 50°C. SBII buffer (50% glycerol supplemented with bromophenol blue) and 1M DTT buffer was added to the cell lysates followed by a 5-minute incubation at 65°C. Whole cell protein samples were resolved by SDS-PAGE electrophoresis and analyzed by Western blot using anti-Rad53 (1:5,000) (Invitrogen) with goat anti-mouse HRP (1:30,000) or by anti-PGK (1:20,000) (Novex) with goat anti-mouse HRP (1:40,000) (Bio-Rad) and ECL prime (GE Healthcare) for visualization.

## Results

### Elevated levels of chromatin-bound PCNA (via *elg1Δ*) promote cohesin binding to DNA in *mcd1* mutant cells

**Fig 1:**
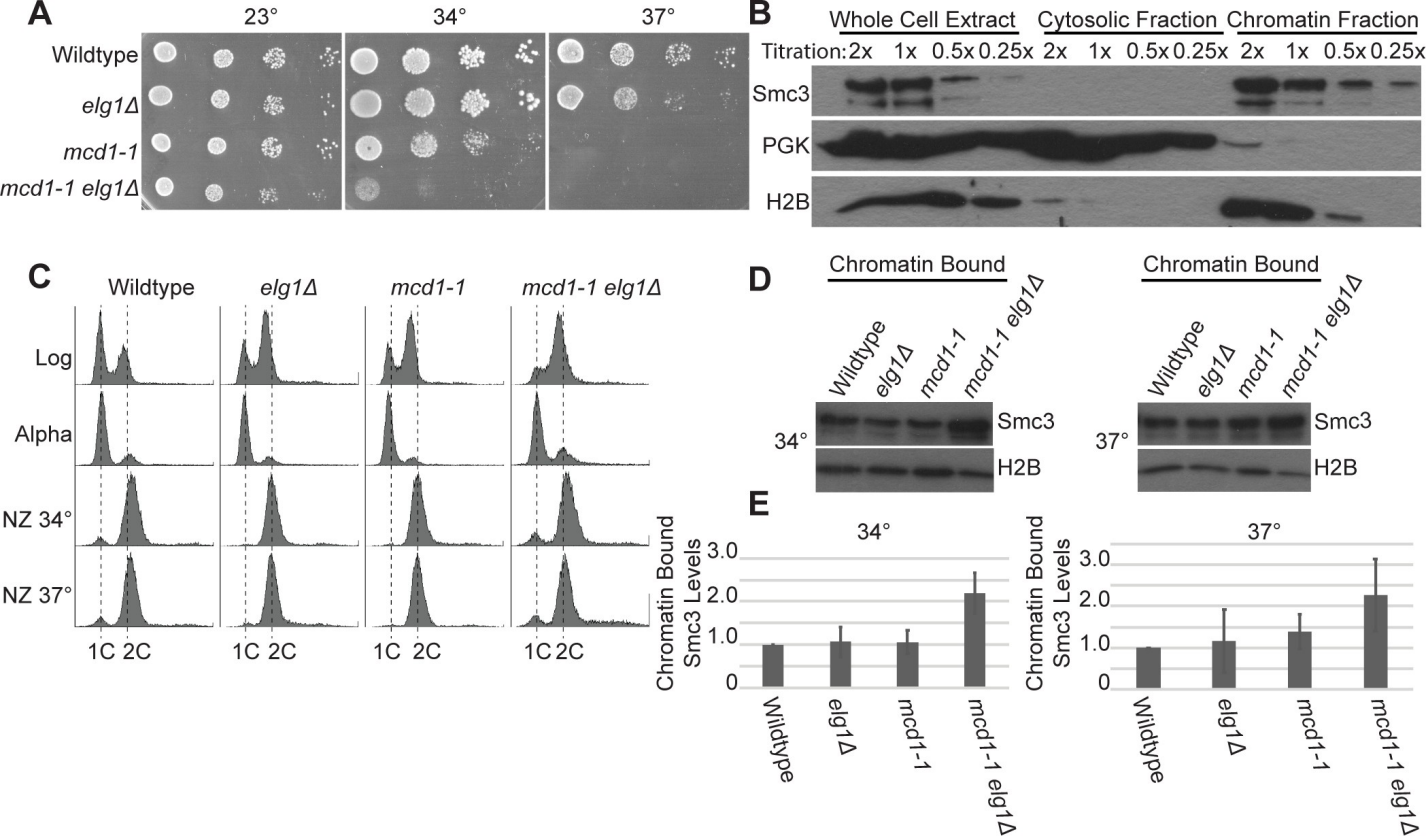
Excess chromatin-bound PCNA (via *elg1Δ*) increases cohesin chromatin levels in *mcd1* mutant cells. [A] 10-fold serial dilution of indicated yeast strains plated on rich medium plates and incubated at 23°C, 34°C, and 37°C for 2 days. [B] Titration and chromatin fractionation of wildtype cells arrested in nocodazole. 1X sample concentration for Smc3, H2B, and PGK indicates samples are within the linear range of detection. [C] DNA content of cells synchronized in G1 with alpha factor, and then shifted to 34°C or 37°C and arrested in pre-anaphase with nocodazole (NZ). [D] Chromatin bound fraction of cells arrested in pre-anaphase. Smc3 and H2B indicate levels of chromatin-bound proteins in the chromatin fraction. [E] Quantification of chromatin bound Smc3 levels, compared to H2B, in the indicated strains. Smc3 enrichment on DNA is based on the ratio of Smc3 to Histone 2B levels and obtained from 3 independent experiments. Error bars represent standard deviation.

PCNA^OE^ (*POL30* expressed from a 2*μ* vector) and *elg1Δ*, both of which result in persistent and increased levels of chromatin-bound PCNA, adversely affect all core cohesin mutant strains tested to date [15, 32, 33, 35, 61, 63]. For instance, *mcd1-1* mutant cells are inviable at the restrictive temperature of 37°C, due to both high cohesion and condensation defects [1, 2], but lose viability at 34°C in response to either PCNA^OE^ or *elg1Δ* [32, 33]. Given that cohesin deposition onto chromatin and cycles of PCNA binding/release are normally coordinated [10, 21, 36], it became important to test whether elevated levels of chromatin-bound PCNA reduce cohesin deposition onto DNA—either through direct competition, reduction of factors that promote cohesin deposition (Scc2,4, RSC or Chl1), or increase in factors (Rad61/WAPL) that dissociate cohesin from chromatin [7, 8, 23, 30, 31, 63, 66–70]. We first independently verified the antagonistic effect that *elg1Δ* imparts on cells harboring *mcd1-1* mutations and indeed found severely exacerbated growth defects (Fig 1A). Next, we validated the efficiency of the Triton X-100-based fractionation procedure [3, 45]. We obtained clear separation of soluble components (using the cytoplasmic factor Phosphoglycerokinase—PGK) from those of chromatin-bound components (Histone 2B) (65). Finally, we optimized Western blot procedures to obtain a linear range of band intensities from which to quantify levels of fractionated chromatin-bound proteins (Fig 1B). To test whether elevated levels of chromatin-bound PCNA reduce cohesin binding to DNA, wildtype, *elg1Δ* and *mcd1-1* single mutant cells, and *mcd1-1 elg1Δ* double mutant cells, all of which express Smc3-3V5 as the sole source of Smc3 protein, were synchronized in G1 and released at either 34°C (the non-permissive temperature for *mcd1-1 elg1Δ* double mutant cells) or 37°C (the non-permissive temperature for *mcd1-1* single mutant cells) in fresh medium containing nocodazole. Cell cycle synchronizations were monitored by flow cytometry (Fig 1C). The resulting pre-anaphase cells were lysed and fractionated prior to assessing for changes in chromatin-bound Smc3, a core component of the cohesin complex [2, 71]. As expected, wildtype and *elg1Δ* single mutant cells display similar levels of chromatin bound Smc3, compared to H2B (Fig 1D and 1E). The results further reveal that *mcd1-1* single mutant cells display similar levels of chromatin-bound Smc3 compared to wildtype cells, even at the non-permissive temperature of 37° (Fig 1D and 1E). Thus, Mcd1 inactivation, which results in cell inviability, cohesion and condensation defects [1, 2], does so in the absence of cohesin dissociation from DNA (Fig 1D and 1E). Surprisingly, *mcd1-1 elg1Δ* double mutant cells display increased levels of chromatin bound cohesin at both non-permissive temperatures of 34° and 37°, compared to that of either wildtype, *elg1Δ* or *mcd1-1* single mutant cells (Fig 1D and 1E). These results that chromatin-bound levels of Smc3 are not reduced in *mcd1-1 elg1Δ* double mutant cells, but instead are increased, reveal that excess chromatin-bound PCNA does not adversely impact cohesin loading onto DNA and negates both direct competition models and recruitment of cohesin releasing factors. The mechanism through which PCNA promotes cohesin deposition onto DNA remains an important issue in chromatin regulation.

### Elevated levels of chromatin-bound PCNA (via *elg1Δ*) promote Smc3 acetylation in *mcd1* mutant cells

**Fig 2:**
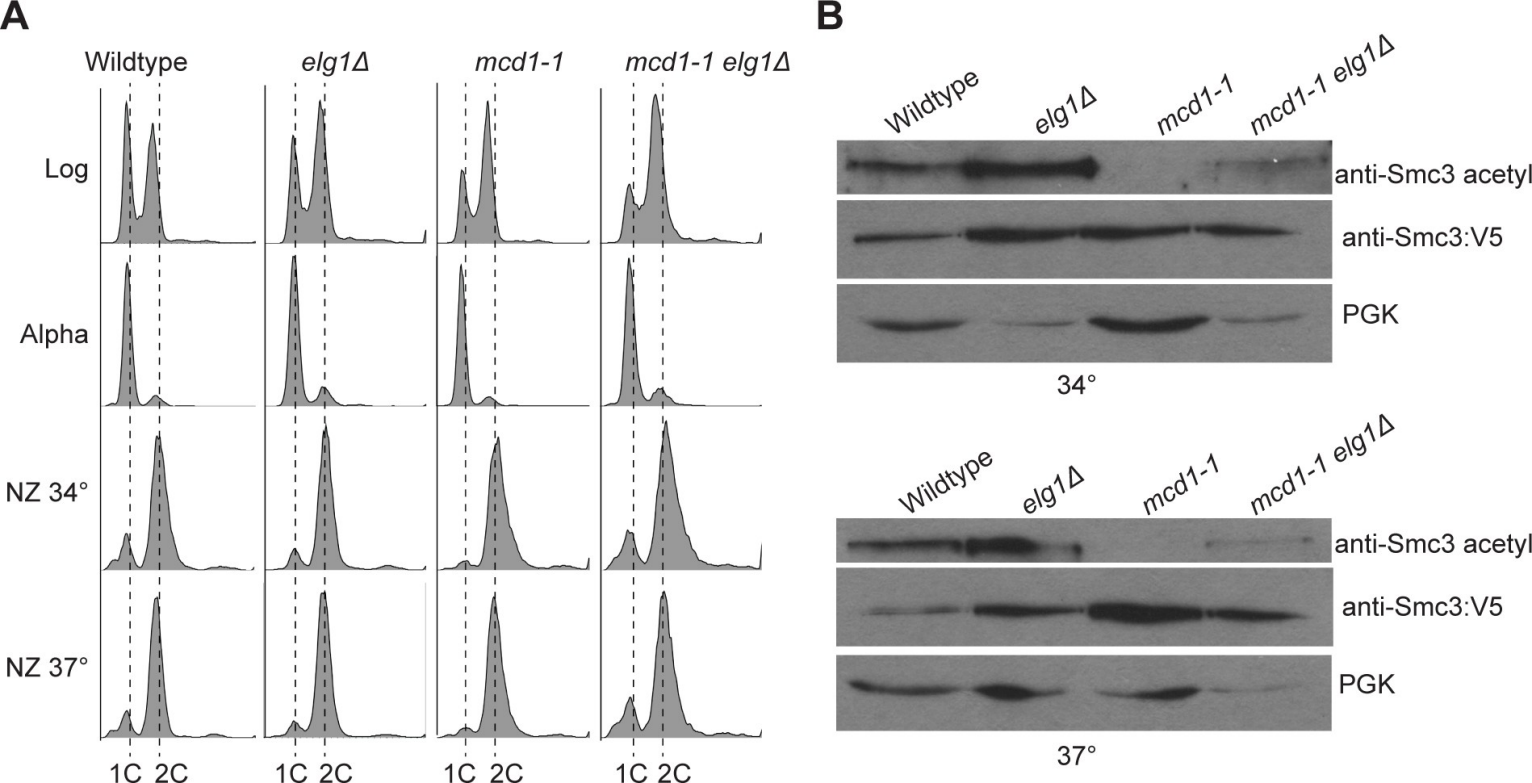
Excess chromatin bound PCNA (via *elg1Δ*) promotes Smc3 acetylation in *mcd1-1* mutant cells. [A] DNA content of log phase cells synchronized in G1, then shifted to 34°C or 37°C and arrested in pre-anaphase. [B] Elevated levels of chromatin-bound PCNA promotes Smc3 acetylation in *mcd1-1* mutant cells. Smc3 was detected by a V5 specific antibody, and acetylated Smc3 was detected by a K112/K113 acetylation antibody. PGK was used as a loading control.

If excess levels of chromatin-bound PCNA (via *elg1Δ*) do not reduce cohesin deposition, PCNA may instead negatively impact acetylation of impaired cohesin to produce the observed cell growth defects. To test this possibility, wildtype, *elg1Δ* and *mcd1-1* single mutant cells, and *mcd1-1 elg1Δ* double mutant cells, all of which express Smc3-3V5 as the sole source of Smc3 function, were synchronized in G1 and released at 34°C or 37°C into fresh medium supplemented with nocodazole (Fig 2A). Protein samples were then assessed for Smc3 acetylation. Wildtype cells and *elg1Δ* single mutant cells contained similar levels of Smc3 acetylation at both temperatures. In contrast, *mcd1-1* single mutant cells exhibited a dramatic decrease in Smc3 acetylation at both temperatures (Fig 2B), despite the retention of both mcd1-1 and Smc3 proteins on DNA (Fig 1E). This provides the first evidence that Mcd1 is critical for Eco1-dependent Smc3 acetylation and thus defines a new role for Mcd1 in promoting cohesion establishment. Importantly, *mcd1-1 elg1Δ* double mutant cells contained increased levels of Smc3 acetylation compared to *mcd1-1* single mutant cells, regardless of a temperature shift to either 34°C or 37°C (Fig 2B, S1 Fig), consistent with prior evidence that elevated levels of PCNA promote both *eco1* mutant cell viability and Eco1-dependent acetylation of Smc3 [10, 15]. In combination, these results reveal that elevated levels of chromatin-bound PCNA, via *elg1Δ*, do not negatively impact either binding or acetylation of cohesin. That elevated levels of chromatin-bound PCNA promotes both the acetylation and deposition of mutant cohesin therefore suggests that the adverse effect of PCNA occurs through some other mechanism.

### Elevated levels of chromatin-bound PCNA (via *2μ POL30*) increases *mcd1* mutant cell sensitivity to DNA damage agents

**Fig 3:**
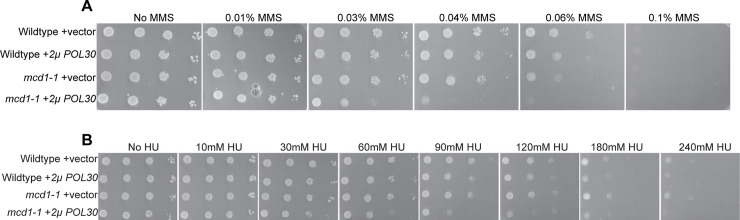
Elevated PCNA levels (via PCNA^OE^) causes DNA damage sensitivity in *mcd1-1* mutant cells. [A] 10-fold serial dilution of indicated yeast strains plated on selective medium plates containing no MMS, 0.01% MMS, 0.03% MMS, 0.04% MMS, 0.06% MMS, or 0.1% MMS, and incubated at 23°C for 3 days. [B] 10-fold serial dilution of indicated yeast strains plated on selective medium plates containing no HU, 10mM HU, 30mM HU, 60mM HU, 90mM HU, 120mM HU, 180mM HU, or 240mM HU, and incubated at 23°C for 3 days.

Given the exclusion of models in which elevated levels of chromatin-bound PCNA (via *elg1Δ*) reduce either cohesin binding to DNA or acetylation, we next hypothesized that PCNA-dependent growth defects of cohesin mutants might involve genomic instability. For instance, it is now well documented that very high levels of PCNA, obtained by *GAL*-based overexpression, renders wildtype cells hypersensitive to genotoxic agents and promotes elevated levels of sister chromatid recombination [63], phenotypes similarly exhibited by *elg1Δ* cells [60, 63, 72–74]. In contrast, studies involving relatively moderate increased levels of PCNA, via 2*μ* plasmids, have thus far failed to produce overt growth defects in wildtype cells [10, 15, 58, 63, 75]. We utilized this same 2*μ* plasmid strategy to ascertain whether a modest increase of chromatin-bound PCNA produces increased sensitivity to genotoxic agents in the context of cohesin mutant cells. Serial dilutions of wildtype and *mcd1-1* mutant cells harboring either *CEN* vector alone or *2μ* vector containing *POL30* (PCNA) were plated on selective media supplemented with increasing concentrations of MMS, and maintained at the permissive temperature of 23°C. Wildtype cells, with or without the *2μ POL30* plasmid, exhibited similar dose-dependent growth defects, but remained viable even at elevated levels of MMS (Fig 3A). *mcd1-1* single mutant cells that harbored the vector alone exhibited growth rates nearly identical to those of wildtype cells across the entire dose range of MMS. In contrast, *mcd1-1* mutant cells that harbored the *2μ POL30* plasmid (PCNA^OE^) produced significant growth defects that coincided with increased MMS levels (Fig 3A). In combination, this result suggests that even moderate PCNA^OE^ adversely impacts genomic integrity, but an effect which is detected here only in cohesin mutant cells sensitized by MMS.

We next tested whether this moderate PCNA^OE^ is sufficient to sensitize *mcd1-1* mutant cells to replication fork destabilizing agents. Hydroxyurea (HU) inhibits RNR function, resulting in depletion of dNTPs and destabilized DNA replication forks [76]. Serial dilutions of wildtype and *mcd1-1* mutant cells, harboring either *CEN* vector alone or with *2μ POL30*, were exposed to various concentrations of HU and maintained at the permissive temperature of 23°C. Wildtype cells, with or without elevated PCNA levels, were largely refractile to HU except at the most extreme concentration of HU, at which PCNA produced a minor growth defect (Fig 3B). Interestingly, *mcd1-1* mutant cells that harbor the vector alone exhibited growth comparable to that of wildtype cells at all concentrations of HU. *mcd1-1* mutant cells that harbor *2μ POL30* plasmid, however, exhibited significant growth defects even at relatively moderate levels of HU (Fig 3B). To assess the effect of elevated levels of chromatin-bound PCNA, in the absence of increased cytosolic levels of PCNA, we also tested the combined effects of *elg1Δ* and DNA damaging agents on wildtype and *mcd1-1* mutant cells. Similar to results obtained from PCNA^OE^, the deletion of *EGL1* from *mcd1-1* cells produced extreme sensitivities to both MMS and HU (S2 Fig). These results are consistent with prior studies that cohesin plays a critical role in stalled replication fork restart as well as DNA damage repair [77–80].

### Elevated levels of chromatin-bound PCNA (via *2μ POL30*) activate the Mec1/ATR DNA damage response pathway and impact cell cycle progression

**Fig 4:**
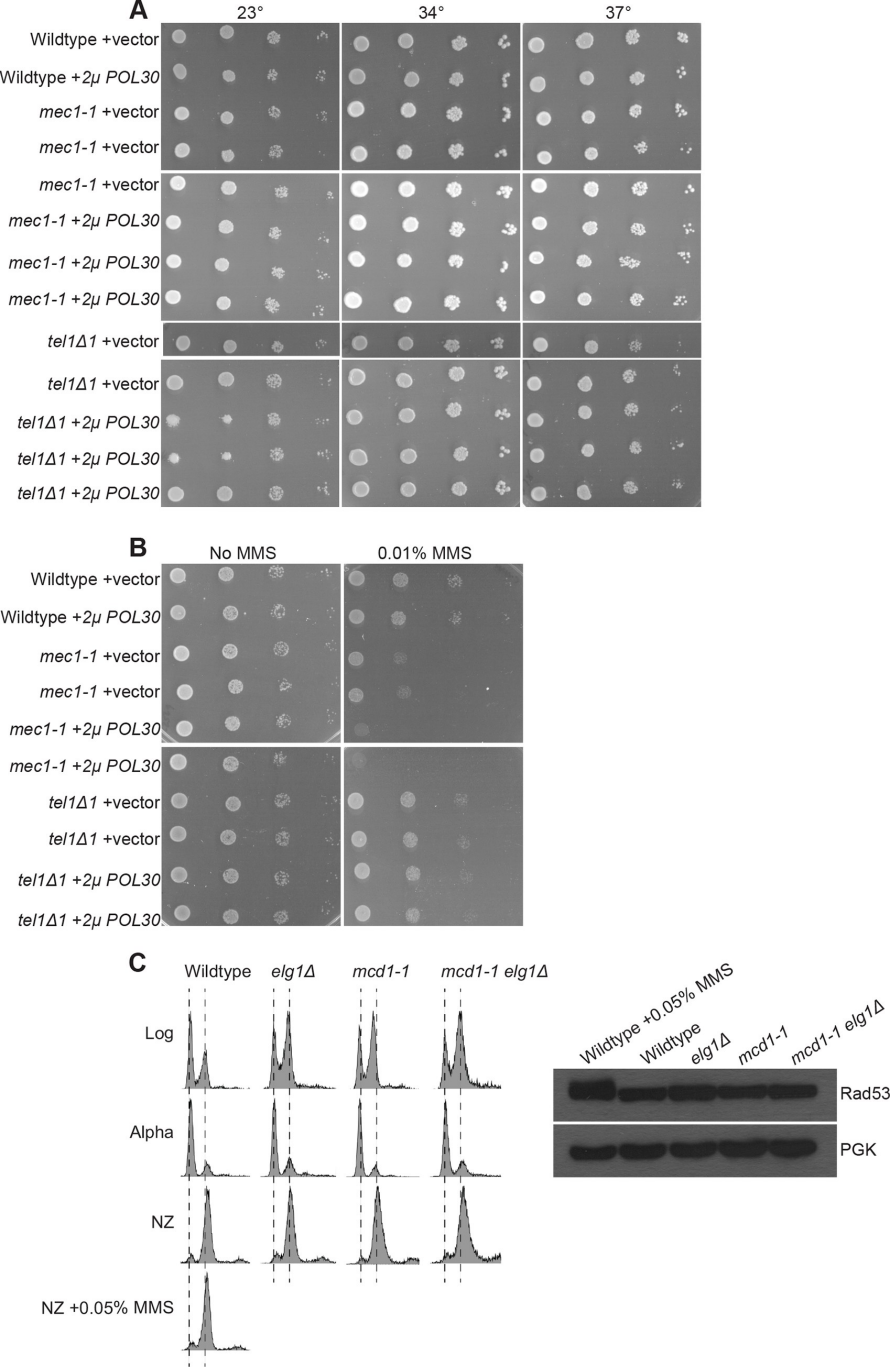
Elevated levels of PCNA sensitizes *mec1-1* mutant cells to MMS. [A] Elevated levels of PCNA does not impact *tel1Δ1* and *mec1-1* mutant cells under high temperature stress. 10-fold serial dilution of indicated yeast strains plated on selective medium plates and incubated at 23°C, 34°C, and 37°C for 2 days. [B] Elevated levels of PCNA specifically sensitizes *mec1-1* mutant cells when exposed to DNA damage. 10-fold serial dilution of indicated yeast strains plated on selective medium plates containing no MMS or 0.01% MMS and incubated at 23°C for 2 days. [C] Rad53 checkpoint is not activated in *mcd1-1 elg1Δ* double mutant cells. DNA content are shown for cells in log phase, synchronized in G1 with alpha factor, and released and shifted to 37°C and arrested in pre-anaphase. Wildtype cells exposed to 0.05% MMS was used as a positive control for Rad53 activation. Rad53 was detected using a Rad53 specific antibody, and PGK was used as a loading control.

The adverse effect of *2μ POL30* and *elg1Δ* on *mcd1-1* mutant cells is consistent with the model that even very moderate levels of PCNA is detrimental to genome maintenance. Mec1 (ATR) and Tel1 (ATM) kinases form parallel DNA damage response pathways [81–84]. To determine which, if either, pathway responds to PCNA^OE^, *tel1Δ1* and *mec1-1* mutant cells were transformed with a CEN vector alone or *2μ* vector with *POL30* and grown on plates maintained at a range of temperatures. Surprisingly, neither *tel1Δ1* nor *mec1-1* mutant cells exhibited any adverse growth rates in response to PCNA^OE^, regardless of the temperature (Fig 4A). We next assessed if either *tel1Δ1* or *mec1-1* mutant cells, sensitized by low doses of MMS, might reveal adverse effects of PCNA^OE^. *tel1Δ1* mutant cells that harbor the *2μ POL30* plasmid exhibited growth nearly identical to that of wildtype cells, despite the presence of MMS. Conversely, *mec1-1* mutant cells that harbor the *2μ POL30* plasmid exhibited significant growth defects in the presence of MMS (Fig 4B).

Phosphorylation of the DNA damage checkpoint kinase Rad53 occurs in a Mec1-dependent manner [85]. Rad53 is not phosphorylated in cells depleted of Elg1 under normal conditions, even though this checkpoint pathway is competent to respond to genotoxic agents [21, 61, 73]. These studies suggest that *mcd1-1 elg1Δ* double mutant cells would similarly not exhibit elevated Rad53 phosphorylation, despite the exacerbated growth defect at the restrictive temperature. To test this prediction, wildtype, both *mcd1-1* and *elg1Δ* single mutants, and *mcd1-1 elg1Δ* double mutant cells were synchronized in G1, washed, and released in nocodazole at 37°C. As a positive control, wildtype cell cultures were split in two, with one population exposed to 0.05% MMS, prior to assessing for Rad53 phosphorylation in all strains. As expected, Rad53 exhibited a clear upward shift in wildtype cells exposed to 0.05% MMS, compared to Rad53 obtained from untreated wildtype and *elg1Δ* single mutant cells, consistent with prior results [61]. Interestingly, neither *mcd1-1* single mutant nor *mcd1-1 elg1Δ* double mutant cells exhibited a shift in Rad53 migration (Fig 4C). These results suggest that increased levels of chromatin-bound PCNA generates genomic instabilities that can activate a Mec1/ATR (but not Tel1/ATM) DNA damage repair pathway but in the absence of Rad53 phosphorylation.

PCNA^OE^ sensitizes *mcd1-1* mutant cells to MMS and HU and also activates the Mec1/ATR intra-S phase checkpoint, suggesting that elevated PCNA levels may adversely impact replication fork progression. Consistent with this hypothesis, mutation of either *ESCO2* (human homolog of yeast *ECO1*) or cohesin subunits result in slowed fork progression in mammalian cells [86–89], although a velocity reduction appears absent in analogous yeast mutant strains [2, 3, 10, 77, 80, 90–92]. It thus became important to test whether elevated levels of chromatin-bound PCNA produces replication stress severe enough to delay S phase progression in *mcd1-1* mutant cells. Log phase wildtype, both *elg1Δ* and *mcd1-1* single mutant cells, and *mcd1-1 elg1Δ* double mutant cells were synchronized in G1, washed, and released into fresh medium supplemented with nocodazole and maintained at the either 32° (restrictive temperature for *mcd1-1 elg1Δ* double mutant cells) or 37°C (restrictive temperature for *mcd1-1* single mutant cells). Aliquots were harvested at 15 minute intervals and cell cycle progression monitored by flow cytometry (S3 Fig). Because cells harboring *ELG1* deletion do not fully synchronize in G1 with alpha factor [32], we analyzed the time point at which at least half of a G1 arrested population progressed to an S-phase state. At 32°C, wildtype and *elg1Δ* mutant cells both reached a half-way point (relatively equal 1N and 2N peaks) between the 30–45 minute time points, while arresting in pre-anaphase in about 75 minutes. *mcd1-1* mutant cells progressed to a mid-way state at the tail end of the time range (45 minutes) required by wildtype and *elg1Δ* mutant cells, and achieved a final preanaphase state in coordination with wildtype and *elg1Δ* mutant cells. At 37°C, which is non-permissive for both *mcd1-1* and *mcd1-1 elg1Δ* mutant cells, *mcd1-1* cells are clearly delayed by almost two time points in progressing to a half-way state (75 minutes), compared to wildtype and *elg1Δ* mutant cells (45–60 minutes) and delayed by a full time point in achieving a preanaphase arrest (90 minutes in *mcd1-1* cells compared to 75 minutes in both wildtype and *elg1Δ* cells. Interestingly, *mcd1-1 elg1Δ* double mutant cells instead both exit G1 and achieve a mid-way state (15 minutes) faster than all other strains at both 32°C and 37°C, but then remain in this mid-way state for an extended period of time of 30 minutes (15–45 minutes) at 37°C, relative to other strains (S3 Fig). These results suggest that elevated levels of chromatin-bound PCNA, in combination with cohesin mutation, adversely impacts replication fork progression.

### The differential impacts of PCNA^OE^ on various cohesin mutant cells

**Fig 5:**
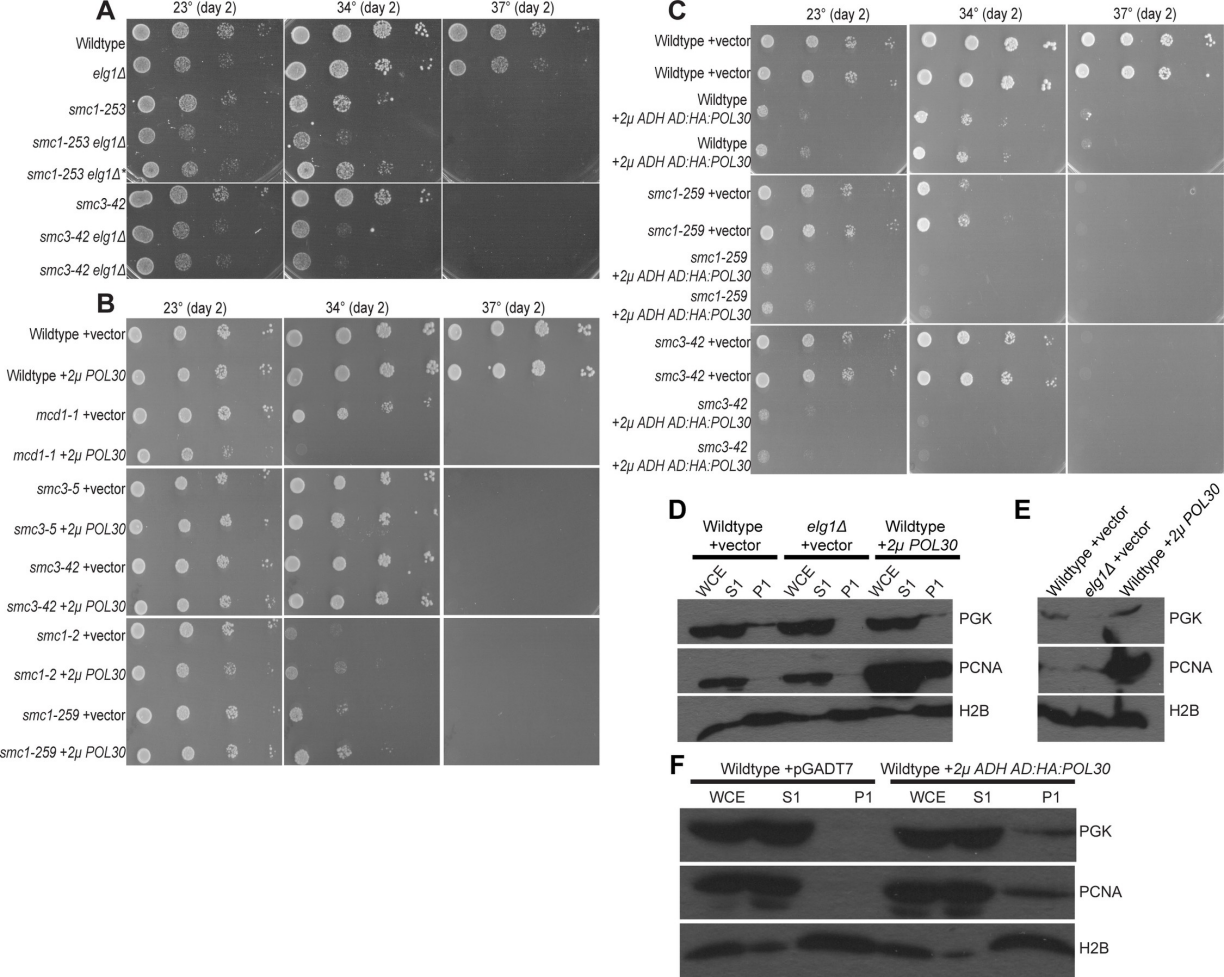
Elevated levels of chromatin-bound PCNA differentially impacts cohesin alleles. [A] *ELG1* deletion exacerbates *smc1-259* and *smc3-42* mutant cells. 10-fold serial dilution of indicated yeast strains plated on rich medium plates and incubated at 23°C, 34°C, and 37°C for 2 days. * indicates a mutated *smc1-259 elg1Δ* strain that displays a resistant to temperature sensitivity [B] PCNA^OE^ from a 2μ plasmid specifically impacts *mcd1-1* mutant cells. 10-fold serial dilution of indicated yeast strains plated on selective medium plates and incubated at 23°C, 34°C, and 37°C for 2 days. [C] Overexpressed PCNA harboring a GAL4 AD and HA N-terminal tag exacerbates cell growth in wildtype cells, *smc1-259*, and *smc3-42* mutant cells. Images of *POL30* in pGADT7 in WT, *smc3-42*, and *smc1-259* mutant cells. 10-fold serial dilution of indicated yeast strains plated on selective medium plates and incubated at 23°C, 34°C, and 37°C for 2 days. [D] Log phase wildtype cells that harbor *2μ POL30* plasmid produce elevated levels of chromatin-bound PCNA, compared to wildtype cells that harbor a vector plasmid and *elg1Δ* single mutant cells. PGK and H2B were used as a loading control and control for chromatin fractionation, respectively. [E] Five times the chromatin fraction of Fig 5D was loaded to visualize chromatin-bound PCNA in wildtype cells. [F] Overexpressed PCNA via a high copy 2*μ* plasmid in log phase wildtype cells results in elevated levels of chromatin-bound PCNA compared to log phase wildtype cells harboring a vector plasmid. PCNA was detected by a PCNA specific antibody. PGK and H2B was used as a loading control and control for chromatin fractionation.

High levels of PCNA (*GAL*-dependent *POL30* overexpression) results in phenotypes that overlap with those obtained by *elg1Δ*, although *elg1Δ* phenotypes are far more robust [63, 73, 74, 93]. It thus became important to ascertain the effect of *elg1Δ* on cohesin mutants to that of a moderate increase (2*μ*-based) in PCNA levels. Our results document that, not only *mcd1-1* cells, but also *smc1-259* and *smc3-42* mutant cells exhibit growth defects in response to *elg1Δ* (Fig 5A), consistent with prior studies [33, 35]. We next tested whether wildtype PCNA (via 2*μ* plasmid) would similarly exacerbate cohesin mutant cell growth. The results reveal that PCNA^OE^ severely exacerbates the temperature sensitivity of *mcd1-1* mutant cells (Fig 5B), consistent with a previous study [15]. Surprisingly, PCNA^OE^ exhibited no adverse effect on either *smc1-259* or *smc3-42* mutant cell growth (Fig 5B). We repeated our analysis on different cohesin alleles. The results show that PCNA^OE^ similarly failed to adversely impact the growth of *smc1-2* and *smc3-5* mutant cells. At first blush, these results appear in stark contrast to those reported by Zhang and colleagues for both *smc1-259* and *smc3-42* mutant cell [15]. Our results, however, are predicated on PCNA expressed from the endogenous promoter in which elevated expression is driven solely by the multi-copy 2*μ* plasmid [10]. In contrast, the Zhang study expressed PCNA using the constitutive and high-expressing ADH promotor in the context of the high copy 2*μ* plasmid (pGADT7), suggesting that the two results may differ due to PCNA expression levels. To test this hypothesis, we cloned *POL30* into pGADT7. The resulting PCNA construct, however, produced a dominant-negative phenotype such that all strains tested, including wildtype cells, exhibited severe growth defects (Fig 5C). We confirmed that both overexpressed PCNA from the multi-copy 2*μ* plasmid and high copy 2*μ* plasmid (pGADT7) resulted in elevated levels of chromatin-bound PCNA (Fig 5D–5F and S4 Fig). Therefore, it is possible that this dominant-negative effect is due to elevated levels of epitope-tagged PCNA as occurs for pGADT7 tagging which is absent in our 2*μ* strategy. Prior studies, for instance, document that endogenous expression of either N- and C-terminus tagged PCNA can result in wildtype cell increased MMS sensitivity [62, 75]. Regardless, our results reveal that *mcd1-1*, in contrast to numerous other cohesin subunit alleles (which exhibit similar conditional growth defects—Fig 5), are hypersensitive to even a moderate increase in wildtype PCNA.

## Discussion

Since the first evidence that cohesion establishment is intimately coupled to DNA replication [10], a wealth of studies document the dependency of Eco1 recruitment by DNA replication fork factors and, furthermore, that replication fork factors regulate sister chromatid tethering reactions [14, 15, 23–25, 29, 32, 33, 35, 36, 94–98]. Of particular interest are RFC complexes that regulate the loading (RFC1^RFC^, Ctf18^RFC^) and unloading (Elg1^RFC^) of PCNA onto DNA [61, 99–101]. Either PCNA overexpression or *ELG1* deletion, both of which result in prolonged and elevated retention of PCNA onto DNA, rescue *eco1* mutant cell growth [10, 15, 32, 33]. Intriguingly, however, elevated levels of chromatin-bound PCNA instead exacerbates the growth defects of the cohesin subunits [15, 33], a surprising phenotype given that the cohesin subunit Smc3 is the target of Eco1 acetylation [12, 13, 102]. *ELG1* deletion is mutagenic and thus may have adverse impacts beyond PCNA unloading while moderate increases in PCNA appear fully tolerated by wildtype cells [58, 60, 63, 73, 75, 103]. Here, we confirm that *ELG1* deletion exacerbates the growth defects present in *mcd1* mutant cells and extend those findings to show that moderate overexpression of wildtype PCNA similarly exerts an adverse growth effect on *mcd1* mutant cell growth. The link between PCNA and cohesin function is complex, however, in that similar expression of PCNA had no impact on a number of other cohesin subunit alleles (*smc3-5*, *smc3-42*, *smc1-295* and *smc1-2*). These findings raise the possibility that cohesin loading onto sister chromatids, which occurs behind DNA polymerase and PCNA [10, 21, 36], may be oriented such that Mcd1 is most proximal to, and thus most impacted by, DNA replication fork components. Interestingly, deletion of *ELG1* (PCNA-unloader), and independent deletion of *CTF18* (PCNA-loader), both exacerbate cohesin mutant cell growth [10, 102]. While our current study was under review, recent findings revealed that dual deletion of *ELG1* and *CTF18* returns to near-normal both chromatin-bound PCNA and cohesion levels, with additional evidence for strand-specific RFC roles in PCNA deposition [21]. Thus, cohesin function is negatively impacted by altering both PCNA dynamics, in either direction, and residency. Additional studies are required to elucidate the multiple mechanisms through which DNA replication fork components, especially involving PCNA, impact cohesin functions.

A second major finding from this study are surprising impacts of elevated levels of chromatin-bound wildtype PCNA on cohesin regulation. Elevated levels of chromatin-bound PCNA that persist well after S phase could antagonize any number of post-replicative activities such as histone deposition, chromatin remodeling, or Okazaki maturation [104, 105]. Given the adverse impact of elevated levels of chromatin-bound PCNA on *mcd1-1* mutant cells, PCNA might conceivably reduce Scc2,Scc4 recruitment and/or subsequent deposition of cohesins onto DNA [8, 23, 66, 106, 107]. An alternate model is that elevated levels of PCNA sequester Eco1 into soluble PCNA pools, precluding sufficient cohesin acetylation. We provide evidence that negate both of these models. Our finding reveals that mutant *mcd1* impedes Smc3 acetylation, a new observation that suggests that Mcd1 supports Eco1-dependent acetylation in a manner yet unknown. Furthermore, our study supports prior findings that elevated chromatin-binding of PCNA improves Smc3 acetylation [10, 15, 21]. More importantly, the finding that increased PCNA promotes cohesin binding onto DNA suggests that factors that promote cohesin deposition (Scc2,4, Chl1, or RSC), or promote cohesin dissociation (Rad61), are themselves sensitive to PCNA levels or modifications [8, 23, 24, 59, 98]. In support of this model are reports that PCNA physically interacts with Chl1, which promotes the recruitment to DNA of both Scc2,4 and cohesin [14, 23, 66, 107]. Intriguingly, increased Smc3 acetylation and cohesin deposition, induced by *elg1Δ*, fails to rescue *mcd1-1* mutant cell growth defects, suggesting that PCNA impacts cohesin functions beyond sister chromatid tethering pathways—as described below.

A third major finding from the current study involves identifying potential mechanisms through which PCNA renders *mcd1-1* mutant cells inviable. It is well known that *elg1Δ* results in long-lived and elevated levels of chromatin-bound PCNA which is highly mutagenic to wildtype cells [73, 74, 93]. High levels of PCNA, achieved by *GAL*-overexpression or coupling *ADH*-overexpression to 2*μ*-based high copy number, similarly produce genomic instabilities [15, 63]. A much more moderate expression system (2*μ* high copy) has thus far shown no adverse effect in wildtype cells [10, 58]. Our findings document that PCNA^OE^ is sufficient to produce growth defects in *mcd1-1* mutant cells, and that this effect is greatly exacerbated by challenging mutant cells with MMS or HU. This intersection between PCNA and cohesin is consistent with prior studies that document that 1) highly elevated levels of PCNA result in genomic instability [63], 2) cohesin are both recruited to stalled replication forks and promote fork restart [77–79, 89], and 3) DNA replication fork protection complexes and cohesin pathways are intimately linked [24, 63, 77, 80, 108]. During the final stages of this study, independent analyses revealed that elevated levels of chromatin-bound PCNA results in hyper-recruitment of mismatch repair factors [109]. Cells that exhibit elevated levels of mismatch repair intermediates normally activate cohesin recruitment pathways to promote efficient DNA repair through homologous recombination [110, 111]. Cells utilize Msh2-Msh3-dependent mismatch repair (MMR) to correct mismatches (including short insertions and deletions) that accumulate during DNA replication [109, 112–114], which increase upon high levels of PCNA expression [109]. However, numerous *mcd1-1 msh3Δ elg1Δ* triple mutant cells were obtained from crossing *msh3Δ* single mutant cells to *mcd1-1 elg1Δ* double mutant cells (S4 Table). Thus, the moderate increase of PCNA used here does not appear to generate long-lived genomic instabilities sufficient to require mismatch repair factor Msh3. Despite this, our findings document that the adverse effect of PCNA^OE^ does result in a Mec1(ATR)-dependent response, versus a Tel1(ATM)-dependent response, but in the absence of Rad53 phosphorylation. The integration of Mec1 sensor in PCNA and cohesin genome instability contrasts those in which double deletion of *MEC1* and *TEL1* were required to reduce Scc1 recruitment to forks under conditions of replication stress [77]. In combination, these findings highlight the complex nature through which Mec1 (and possibly Tel1) respond to replication stressors (such as PCNA^OE^), which are revealed only under conditions of cohesin mutation and/or genotoxic agents.

**Fig 6:**
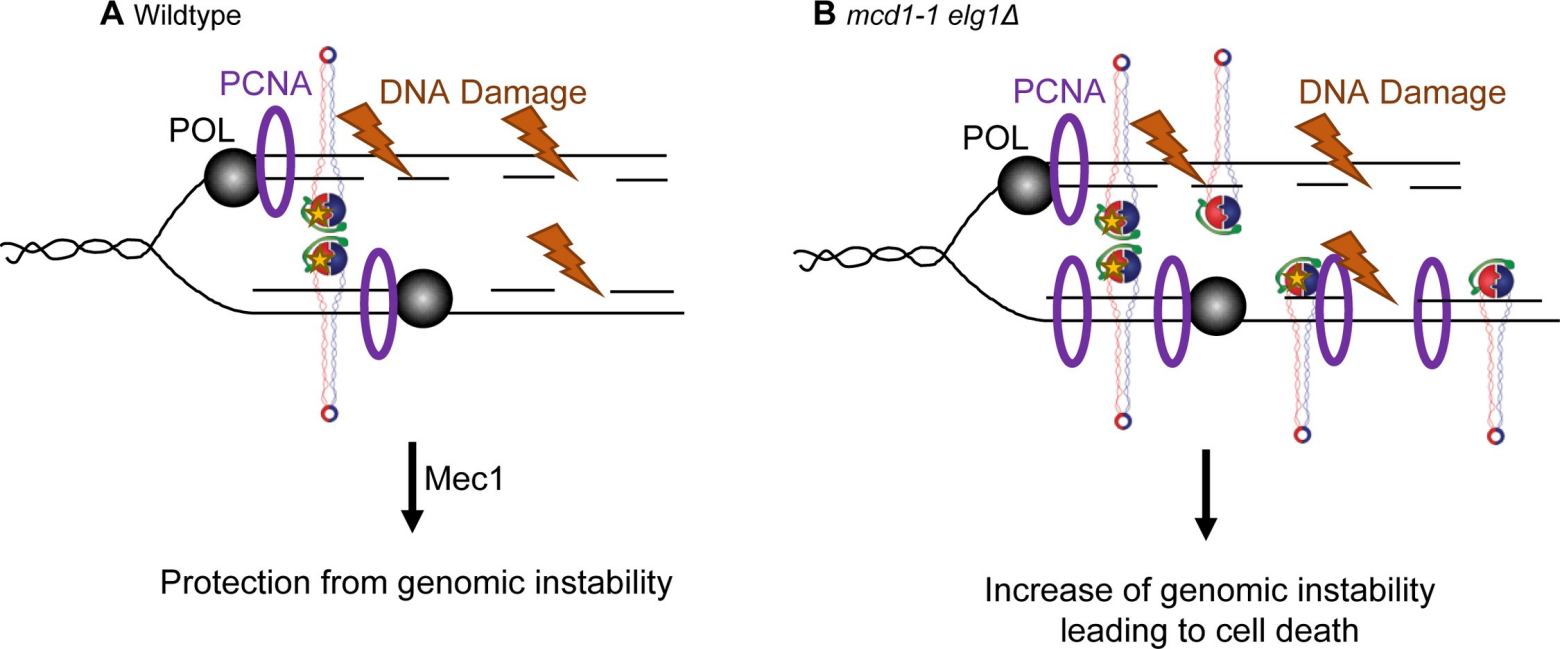
Cohesins are necessary to resolve genomic instabilities caused by elevated levels of PCNA. [A] In wildtype cells, a functional Elg1 removes PCNA from behind the replication fork. If DNA damage occurs, the damage is resolved in a Mec1-dependent manner. Proper DNA repair and protection from genomic instability results in cell viability. [B]. In *mcd1-1 elg1Δ* double mutant cells, loss of Elg1 results in the retention of chromatin-bound PCNA. The combination of misregulated PCNA and a mutant cohesin (which exhibits reduced acetylation) may result in unrepaired DNA and unresolved genomic stability, leading to cell death.

A confluence of findings document that 1) cohesins are deposited during S phase onto nascent sister chromatids [8, 10, 65, 115–120], 2) cohesins are critical for replication fork restart and repair [77–79, 88], 3) Eco1-dependent cohesion establishment is intimately coupled to PCNA and the DNA replication fork [10, 15, 16, 25–27, 121–123], and 4) PCNA^OE^ and *elg1Δ* both adversely impact cohesin mutant cell growth. Clearly, PCNA^OE^ (at even very moderate levels) and *elg1Δ* adversely impact cohesin mutant cell growth, an effect exacerbated by genotoxic agents. We thus posit a model that may explain why elevated levels of PCNA reduce *mcd1-1* mutant cell growth (Fig 6). In wildtype cells, PCNA is removed from chromatin by Elg1 and proper cohesin deposition and acetylation occur to establish sister chromatid cohesion. Elevated levels of chromatin-bound PCNA result in minor genomic instabilities that likely impact replication fork progression, but at a level insufficient for Mec1 to promote Rad53 phosphorylation. Cohesin mutant cells are unable to counter the adverse effects of PCNA—which eventually leads to cell death.

## Supporting information

S1 Fig Elevated levels of PCNA promote Eco1-dependent cohesin acetylation. Second biological iteration of Fig 2. Smc3 was detected by a V5 specific antibody, and acetylated Smc3 was detected by a K112/K113 acetylation antibody. PGK was used as a loading control.
(PDF)

S2 Fig Elevated chromatin-bound PCNA (via *elg1Δ*) causes DNA damage sensitivity in *mcd1-1* mutant cells. [A] 10-fold serial dilution of indicated yeast strains plated on selective medium plates containing no MMS, 0.03% MMS, 0.06% MMS, or 0.1% MMS, and incubated at 23°C for 3 days. [B] 10-fold serial dilution of indicated yeast strains plated on selective medium plates containing no HU, 60mM HU, 120mM HU, or 240mM HU, and incubated at 23°C for 3 days.
(PDF)

S3 Fig Excess chromatin bound PCNA does not cause a S-phase checkpoint activation in wildtype or *mcd1-1* mutant cells. DNA content of log phase cells synchronized in G1. Temperature was then shifted to either 32°C or 37°C and cells released into media supplemented with nocodazole. Samples were collected every 15 minutes.
(PDF)

S4 Fig PCNA overexpressed from a multi-copy 2μ plasmid and a high copy 2*μ* plasmid results in elevated levels of chromatin-bound PCNA. [A] Second biological replicate of Fig 5D. Wildtype cells harboring a *2μ POL30* plasmid results in elevated levels of chromatin-bound PCNA compared to wildtype cells harboring a vector plasmid and *elg1Δ* single mutant cells. PCNA was detected by a PCNA specific antibody. PGK and H2B was used as a loading control and control for chromatin fractionation. [B] Five times the chromatin fraction was loaded to visualize chromatin-bound PCNA in wildtype cells. [C] Second biological replicate of Fig 5F. Overexpressed PCNA via a high copy 2*μ* plasmid results in elevated levels of chromatin-bound PCNA compared to wildtype cells harboring a vector plasmid. PCNA was detected by a PCNA specific antibody. PGK and H2B was used as a loading control and control for chromatin fractionation.
(PDF)

S1 Table Reagents used in this study. (DOCX)

S2 Table Strains used in this study. (DOCX)

S3 Table Plasmids used in this study. (DOCX)

S4 Table *msh3Δ* does not result in cell spore lethality in *mcd1-1 elg1Δ* double mutant cells. Dissection of *mcd1-1 elg1Δ Smc3*:*3V5* mated with *msh3Δ*. *mcd1-1 elg1Δ msh3Δ* triple mutant strains are obtained at the expected frequency.
(DOCX)
