# Supplementary figures and images for: PCNA antagonizes cohesin-dependent roles in genomic stability
Source: PLoS One. 2020 Oct 19;15(10):e0235103. doi: 10.1371/journal.pone.0235103 (PMC7571713; doi:10.1371/journal.pone.0235103)

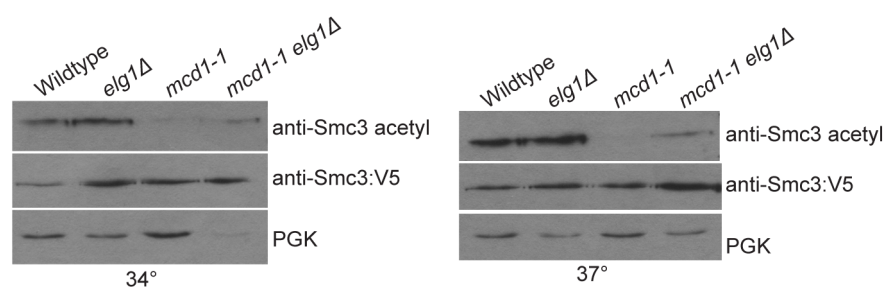

Supplement: S1 Fig — Second biological iteration of Fig 2. Smc3 was detected by a V5 specific antibody, and acetylated Smc3 was detected by a K112/K113 acetylation antibody. PGK was used as a loading control. (PDF) [file pone.0235103.s001.pdf]

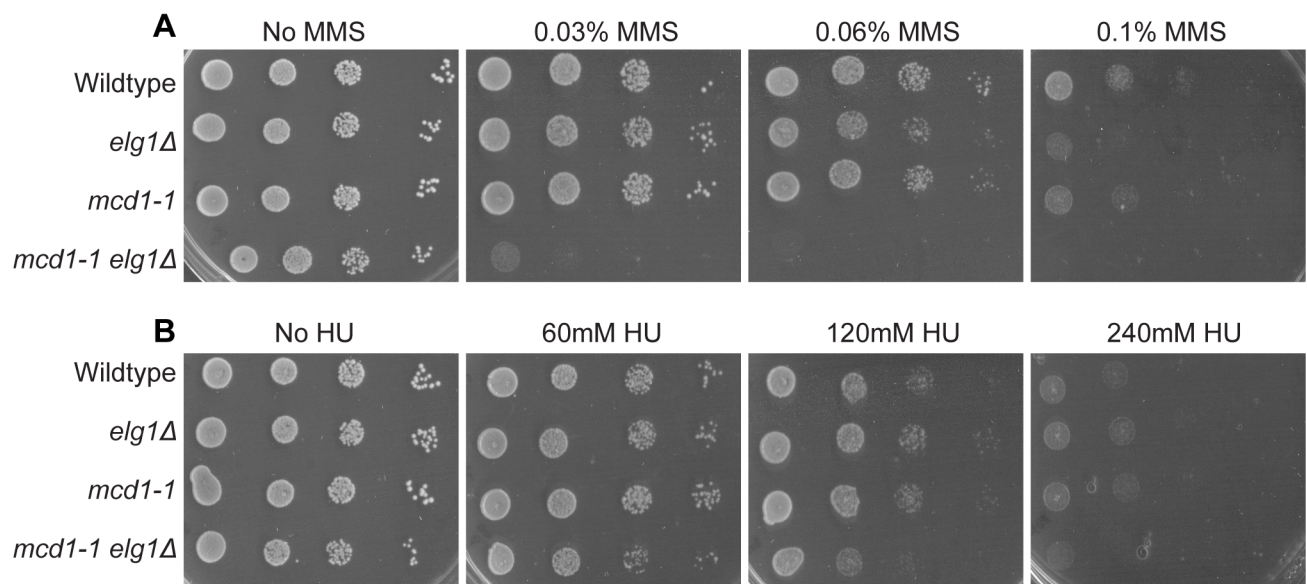

Supplement: S2 Fig — [A] 10-fold serial dilution of indicated yeast strains plated on selective medium plates containing no MMS, 0.03% MMS, 0.06% MMS, or 0.1% MMS, and incubated at 23°C for 3 days. [B] 10-fold serial dilution of indicated yeast strains plated on selective medium plates containing no HU, 60mM HU, 120mM HU, or 240mM HU, and incubated at 23°C for 3 days. (PDF) [file pone.0235103.s002.pdf]

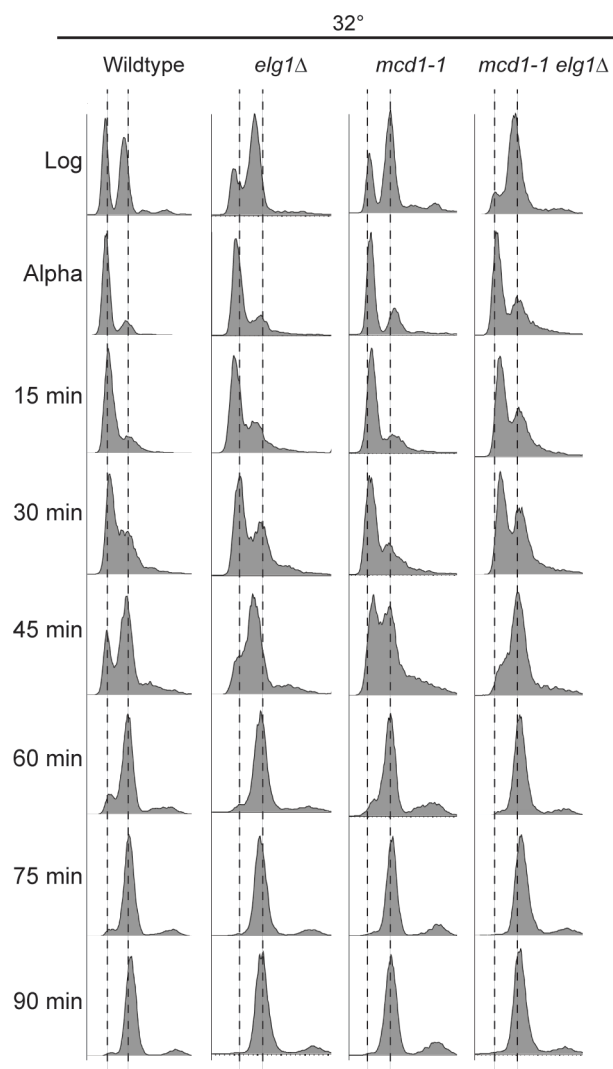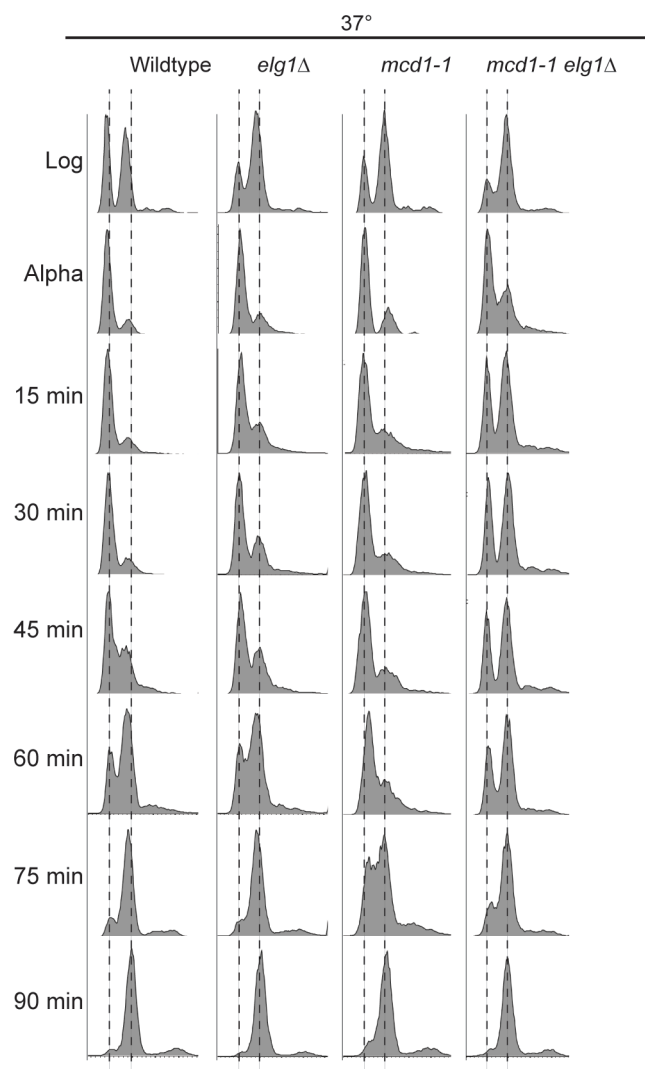

Supplement: S3 Fig — DNA content of log phase cells synchronized in G1. Temperature was then shifted to either 32°C or 37°C and cells released into media supplemented with nocodazole. Samples were collected every 15 minutes. (PDF) [file pone.0235103.s003.pdf]

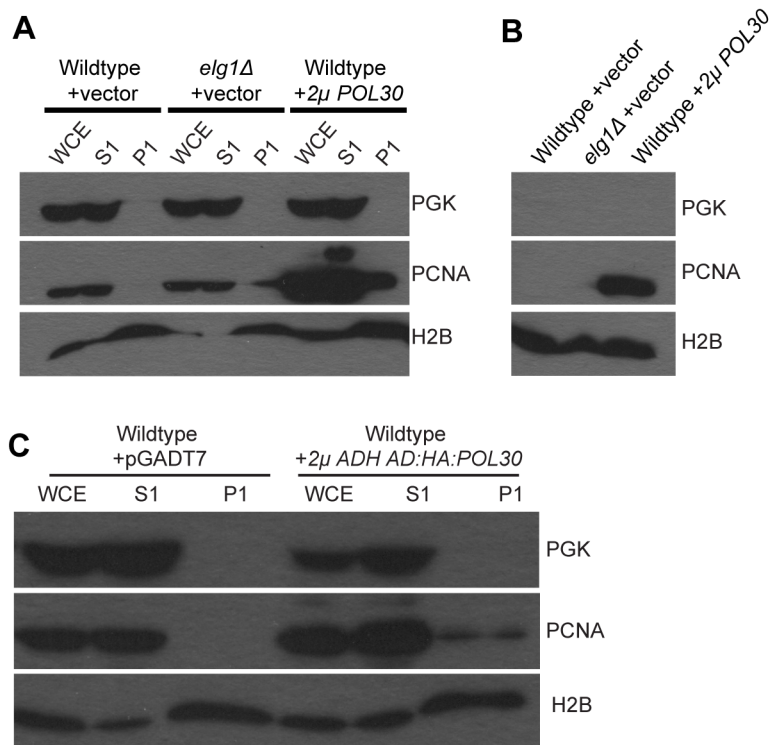

Supplement: S4 Fig — [A] Second biological replicate of Fig 5D. Wildtype cells harboring a 2μ POL30 plasmid results in elevated levels of chromatin-bound PCNA compared to wildtype cells harboring a vector plasmid and elg1Δ single mutant cells. PCNA was detected by a PCNA specific antibody. PGK and H2B was used as a loading control and control for chromatin fractionation. [B] Five times the chromatin fraction was loaded to visualize chromatin-bound PCNA in wildtype cells. [C] Second biological replicate of Fig 5F. Overexpressed PCNA via a high copy 2μ plasmid results in elevated levels of chromatin-bound PCNA compared to wildtype cells harboring a vector plasmid. PCNA was detected by a PCNA specific antibody. PGK and H2B was used as a loading control and control for chromatin fractionation. (PDF) [file pone.0235103.s004.pdf]
