# Supplementary material for: PCNA antagonizes cohesin-dependent roles in genomic stability
Source: PLoS One. 2020 Oct 19;15(10):e0235103. doi: 10.1371/journal.pone.0235103 (PMC7571713; doi:10.1371/journal.pone.0235103)
Supplement: S1 Table — (DOCX) [file pone.0235103.s005.docx]

| S1 Table: Reagents used in this study | | | | |
| --- | --- | --- | --- | --- |
| Reagent type | **Designation** | **Source or reference** | **Identifiers** | **Additional Information** |
|  |  |  |  |  |
| Chemical compound | Alpha Factor | Zymo | Y1001 |  |
| Chemical compound | Nocodozole | Sigma | M1404 |  |
| Chemical compound | Methyl methanesulfonate | ACROS | 66-27-3 | noted as MMS in text |
| Chemical compound | Hydroxyurea | Sigma | H8627 | noted as HU in text |
| Chemical compound | Protease inhibitor cocktail | Sigma | P8215 |  |
| Chemical compound | ECL Prime | GE Healthcare | RPN2232 |  |
| Chemical compound | Glass beads | BioSpec | 11079105 |  |
| Chemical compound | IGEPAL-630 | Sigma | I-3021 |  |
| Antibody | Anti-Rad53 | Abcam | ab104232 | WB: 1:5,000 |
| Antibody | Rabbit anti-PCNA | Abcam | ab221196 | WB: 1:1,000 |
| Antibody | Mouse anti-PGK | Novex | 459250 | WB: 1:20,000 |
| Antibody | Rabbit anti-H2B | Abcam | ab188291 | WB: 1:80,000 |
| Antibody | Mouse anti-V5 | Invitorgen | R960-25 | WB: 1:40,000 |
| Antibody | Mouse anti-SMC3 K112/K113 acetylation | Dr. Katsuhiko Shirahige |  | WB: 1:1,000 |
| Antibody | Goat anti-rabbit HRP | Bio-Rad | 170-6515 | WB: 1:40,000 |
| Antibody | Goat anti-mouse HRP | Bio-Rad | 170-6516 | WB: 1:10,000 (anti-acetyl) or 1:40,000 (V5 and PGK) |
| Genetic Reagent | *Saccharomyces cerevisiae* | this paper | Yeast strains | Supplementary table 2 |
| Genetic Reagent | *Escherichia coli* plasmids | this paper | DNA Plasmids | Supplementary table 3 |
