# Supplementary material for: PCNA antagonizes cohesin-dependent roles in genomic stability
Source: PLoS One. 2020 Oct 19;15(10):e0235103. doi: 10.1371/journal.pone.0235103 (PMC7571713; doi:10.1371/journal.pone.0235103)
Supplement: S2 Table — (DOCX) [file pone.0235103.s006.docx]

| S2 Table: Strains used in this study |
| --- |
|  |
| Strain Genotype Reference |
| DLY285 *MATa mec1-1:HIS ura3 leu2 trp1 his3* *Paulovich, 1995* |
| EU3430-9A *MATa SMC3-3V5-HIS3MX leu2-3, 112 his3-11, 15 lys2-801 trp1-1 bar1 GAL+* *Unal, 2008* |
| K699 *MATa ade 2-1 his 3-11,15 leu 2-3,112 trp 1-1 ura3 can1-100 GAL psi+* *Schwob, 1994* |
| K5824 *MATa ade 2-1 his 3-11,15 leu 2-3,112 trp 1-1 ura3 can1-100 smc3-42* *Stead, 2003* |
| K6013 *MATa ade 2-1 his 3-11,15 leu 2-3,112 trp 1-1 ura3 can1-100 GAL psi+ smc1-259* *Stead, 2003* |
| YBS2021 *MATa ade 2-1 his 3-11,15 leu 2-3,112 trp 1-1 ura3-1 mcd1-1 Net1:GFP:KAN* This study |
| YDM884 *MATa tel1∆1::HIS3 ura3-52 his3∆200 ade2-101 leu2∆1 lys2-801 trp1∆1 Morrow, 1995* |
| YMM433 *MATa ade 2-1 his 3-11,15 leu 2-3,112 trp 1-1 ura3-1 can1-100 smc1-2* This study |
| YMM435 *MATa ade 2-1 his 3-11,15 leu 2-3,112 trp 1-1 ura3-1 can1-100 smc3-5* This study |
| YCZ044 *MATα ade 2-1 his 3-11,15 leu 2-3,112 trp 1-1 ura3-1* This study |
| YCZ143 *MATα ade 2-1 his 3-11,15 leu 2-3,112 trp 1-1 ura3-1 elg1::KAN* This study |
| YCZ144 *MATa ade 2-1 his 3-11,15 leu 2-3,112 trp 1-1 ura3-1 mcd1-1* This study |
| YCZ147 *MATa ade 2-1 his 3-11,15 leu 2-3,112 trp 1-1 ura3-1* This study |
| YCZ225 *MATa ade 2-1 his 3-11,15 leu 2-3,112 trp 1-1 ura3-1 mcd1-1 2µ POL30:URA* This study |
| YCZ249 *MATa ade 2-1 his 3-11,15 leu 2-3,112 trp 1-1 ura3-1 KAN:ECO1, elg1∆::TRP* This study |
| YCZ262 *MATα ade 2-1 his 3-11,15 leu 2-3,112 trp 1-1 ura3-1 SMC3:V5:HIS* This study |
| YCZ280 *MATα ade 2-1 his 3-11,15 leu 2-3,112 trp 1-1 ura3-1 elg1∆::TRP SMC3:V5:HIS* This study |
| YCZ407 *MATa ade 2-1 his 3-11,15 leu 2-3,112 trp 1-1 ura3-1 SMC3:V5:HIS* This study |
| YCZ408 *MATa ade 2-1 his 3-11,15 leu 2-3,112 trp 1-1 ura3-1 mcd1-1 elg1∆::TRP SMC3:V5:HIS* This study |
| YCZ421 *MATα ade 2-1 his 3-11,15 leu 2-3,112 trp 1-1 ura3-1 mcd1-1 elg1∆::TRP SMC3:V5:HIS* This study |
| YCZ425 *MATa ade 2-1 his 3-11,15 leu 2-3,112 trp 1-1 ura3-1 mcd1-1 SMC3:V5:HIS* This study |
| YCZ428 *MATa ade 2-1 his 3-11,15 leu 2-3,112 trp 1-1 ura3-1 elg1∆::TRP SMC3:V5:HIS* This study |
| YCZ465 *MATa ade 2-1 his 3-11,15 leu 2-3,112 trp 1-1 ura3-1 2µ POL30:URA* This study |
| YCZ470 *MATa ade 2-1 his 3-11,15 leu 2-3,112 trp 1-1 ura3-1 elg1::KAN CEN vector:URA* This study |
| YCZ474 *MATa ade 2-1 his 3-11,15 leu 2-3,112 trp 1-1 ura3-1 mcd1-1 CEN vector:URA* This study |
| YCZ477 *MATa ade 2-1 his 3-11,15 leu 2-3,112 trp 1-1 ura3-1 CEN vector:URA* This study |
| YCZ530 *MATa ade 2-1 his 3-11,15 leu 2-3,112 trp 1-1 ura3-1 can1-100 smc1-2 CEN vector:URA* This study |
| YCZ532 *MATa ade 2-1 his 3-11,15 leu 2-3,112 trp 1-1 ura3-1 can1-100 smc1-2 2µ POL30:URA* This study |
| YCZ534 *MATa ade 2-1 his 3-11,15 leu 2-3,112 trp 1-1 ura3-1 can1-100 smc3-5 CEN vector:URA* This study |
| YCZ536 *MATa ade 2-1 his 3-11,15 leu 2-3,112 trp 1-1 ura3-1 can1-100 smc3-5 2µ POL30:URA* This study |
| YCZ559 *MATa ade 2-1 his 3-11,15 leu 2-3,112 trp 1-1 ura3 can1-100 smc3-42 CEN vector:URA* This study |
| YCZ561 *MATa ade 2-1 his 3-11,15 leu 2-3,112 trp 1-1 ura3 can1-100 smc3-42 2µ POL30:URA* This study |
| YCZ563 *MATa ade 2-1 his 3-11,15 leu 2-3,112 trp 1-1 ura3 can1-100 GAL psi+ smc1-259 CEN vector:URA* This study |
| YCZ565 *MATa ade 2-1 his 3-11,15 leu 2-3,112 trp 1-1 ura3 can1-100 GAL psi+ smc1-259 2µ POL30:URA* This study |
| YCZ567 *MATa mec1-1:HIS ura3 leu2 trp1 his3 CEN vector:URA* This study |
| YCZ568 *MATa mec1-1:HIS ura3 leu2 trp1 his3 CEN vector:URA* This study |
| YCZ575 *MATa mec1-1:HIS ura3 leu2 trp1 his3 CEN vector:URA* This study |
| YCZ569 *MATa mec1-1:HIS ura3 leu2 trp1 his3 2µ POL30:URA* This study |
| YCZ570 *MATa mec1-1:HIS ura3 leu2 trp1 his3 2µ POL30:URA* This study |
| YCZ576 *MATa mec1-1:HIS ura3 leu2 trp1 his3 2µ POL30:URA* This study |
| YCZ571 *MATa tel1∆1::HIS3 ura3-52 his3∆200 ade2-101 leu2∆1 lys2-801 trp1∆1 CEN vector:URA* This study |
| YCZ572 *MATa tel1∆1::HIS3 ura3-52 his3∆200 ade2-101 leu2∆1 lys2-801 trp1∆1 CEN vector:URA* This study |
| YCZ577 *MATa tel1∆1::HIS3 ura3-52 his3∆200 ade2-101 leu2∆1 lys2-801 trp1∆1 CEN vector:URA* This study |
| YCZ573 *MATa tel1∆1::HIS3 ura3-52 his3∆200 ade2-101 leu2∆1 lys2-801 trp1∆1 2µ POL30:URA* This study |
| YCZ574 *MATa tel1∆1::HIS3 ura3-52 his3∆200 ade2-101 leu2∆1 lys2-801 trp1∆1 2µ POL30:URA* This study |
| YCZ578 *MATa tel1∆1::HIS3 ura3-52 his3∆200 ade2-101 leu2∆1 lys2-801 trp1∆1 2µ POL30:URA* This study |
| YCZ662 *MATa ade 2-1 his 3-11,15 leu 2-3,112 trp 1-1 ura3-1 2µ ADH:GAL4AD:HA Vector:LEU* This study |
| YCZ663 *MATa ade 2-1 his 3-11,15 leu 2-3,112 trp 1-1 ura3-1 2µ ADH:GAL4AD:HA Vector:LEU* This study |
| YCZ664 *MATa ade 2-1 his 3-11,15 leu 2-3,112 trp 1-1 ura3-1 2µ ADH:GAL4AD:HA:POL30:LEU* This study |
| YCZ665 *MATa ade 2-1 his 3-11,15 leu 2-3,112 trp 1-1 ura3-1 2µ ADH:GAL4AD:HA:POL30:LEU* This study |
| YCZ666 *MATa ade 2-1 his 3-11,15 leu 2-3,112 trp 1-1 ura3-1 smc1-259 2µ ADH:GAL4AD:HA Vector:LEU* This study |
| YCZ667 *MATa ade 2-1 his 3-11,15 leu 2-3,112 trp 1-1 ura3-1 smc1-259 2µ ADH:GAL4AD:HA Vector:LEU* This study |
| YCZ668 *MATa ade 2-1 his 3-11,15 leu 2-3,112 trp 1-1 ura3-1 smc1-259 2µ ADH:GAL4AD:HA:POL30:LEU* This study |
| YCZ669 *MATa ade 2-1 his 3-11,15 leu 2-3,112 trp 1-1 ura3-1 smc1-259 2µ ADH:GAL4AD:HA:POL30:LEU* This study |
| YCZ670 *MATa ade 2-1 his 3-11,15 leu 2-3,112 trp 1-1 ura3-1 smc3-42 2µ ADH:GAL4AD:HA Vector:LEU* This study |
| YCZ671 *MATa ade 2-1 his 3-11,15 leu 2-3,112 trp 1-1 ura3-1 smc3-42 2µ ADH:GAL4AD:HA Vector:LEU* This study |
| YCZ672 *MATa ade 2-1 his 3-11,15 leu 2-3,112 trp 1-1 ura3-1 smc3-42 2µ ADH:GAL4AD:HA:POL30:LEU* This study |
| YCZ673 *MATa ade 2-1 his 3-11,15 leu 2-3,112 trp 1-1 ura3-1 smc3-42 2µ ADH:GAL4AD:HA:POL30:LEU* This study |
| YCZ702 *MATa/α ade 2-1 his 3-11,15 leu 2-3,112 trp 1-1 ura3-1 smc1-259 elg1∆::KAN* This study |
| YCZ706 *MATa ade 2-1 his 3-11,15 leu 2-3,112 trp 1-1 ura3-1 smc3-42 elg1∆::KAN* This study |
| YCZ707 *MATa ade 2-1 his 3-11,15 leu 2-3,112 trp 1-1 ura3-1 smc3-42 elg1∆::KAN* This study |
| YCZ728 *MATa ade 2-1 his 3-11,15 leu 2-3,112 trp 1-1 ura3-1 smc1-259 elg1∆::KAN* This study |
| YCZ730 *MATα ade 2-1 his 3-11,15 leu 2-3,112 trp 1-1 ura3-1 smc1-259 elg1∆::KAN* This study |
| YCZ778 *MATa his3-1 leu2-0 lys2-0 ura3-0 msh3∆::KAN Baudin, 1993* *and Wach, 1994* |
| YCZ799 *MATa/α ade 2-1 his 3-11,15 leu 2-3,112 trp 1-1 ura3-1 his3-1 leu2-0 lys2-0 ura3-0 msh3∆::KAN elg1∆::TRP mcd1-1* This study |
| All strains are in the W303 background except for DLY285, YCZ567, YCZ568, YCZ569, YCZ570, YCZ575, YCZ576 (A364a), EU3430-9A (A364a), YDM884, YCZ571, YCZ572, YCZ573, YCZ574, YCZ577, YCZ578 (A364a), and YCZ778 (BY4743) and YCZ799 (W303/BY4743 diploid) |
