# Supplementary material for: PCNA antagonizes cohesin-dependent roles in genomic stability
Source: PLoS One. 2020 Oct 19;15(10):e0235103. doi: 10.1371/journal.pone.0235103 (PMC7571713; doi:10.1371/journal.pone.0235103)
Supplement: S3 Table — (DOCX) [file pone.0235103.s007.docx]

| S3 Table: Plasmids used in this study |
| --- |
|  |
| Plasmid Genotype Reference |
| pBS99 *2u POL30:URA* *Skibbens et al., 1999* |
| pRS316 CEN Vector *CEN:URA* *Sikorski,. and Hieter., 1989* |
| pGADT7 *2µ* Vector *ADH:HA:LEU* *TakaraBio #630442* |
| pCZ058 *2µ* *ADH:HA:POL30:LEU* *This study* |
