# Supplementary material for: PCNA antagonizes cohesin-dependent roles in genomic stability
Source: PLoS One. 2020 Oct 19;15(10):e0235103. doi: 10.1371/journal.pone.0235103 (PMC7571713; doi:10.1371/journal.pone.0235103)
Supplement: S4 Table — Dissection of mcd1-1 elg1Δ Smc3:3V5 mated with msh3Δ. mcd1-1 elg1Δ msh3Δ triple mutant strains are obtained at the expected frequency. (DOCX) [file pone.0235103.s008.docx]

| S4 Table: Yeast tetrad dissections of *mcd1-1 elg1Δ Smc3:3V5* x *msh3Δ* | | |
| --- | --- | --- |
| Genotype | **Expected** | **Observed** |
| Wildtype | 5 | 0 |
| *mcd1-1* | 5 | 5 |
| *elg1Δ* | 5 | 3 |
| *msh3Δ* | 5 | 0 |
| Smc3:3V5 | 5 | 1 |
| *mcd1-1 elg1Δ* | 5 | 4 |
| *mcd1-1* Smc3:3V5 | 5 | 2 |
| *mcd1-1 msh3Δ* | 5 | 0 |
| *elg1Δ* Smc3:3V5 | 5 | 3 |
| *elg1Δ msh3Δ* | 5 | 3 |
| *msh3Δ* Smc3:3V5 | 5 | 0 |
| *mcd1-1 elg1Δ* Smc3:3V5 | 5 | 0 |
| *mcd1-1 elg1Δ msh3Δ* | 5 | 5 |
| *mcd1-1 msh3Δ* Smc3:3V5 | 5 | 2 |
| *elg1Δ msh3Δ* Smc3:3V5 | 5 | 3 |
| *mcd1-1 elg1Δ msh3Δ* Smc3:3V5 | 5 | 2 |
| DEAD | 0 | 39 |
|  |  |  |
| TOTAL | 76 | 72 |
